# Upper-Bound Electromagnetic Performance of Substrate-Free Epidermal Tattoo Antennas for UHF Applications

**DOI:** 10.3390/s26031011

**Published:** 2026-02-04

**Authors:** Adina Bianca Barba, Alessio Mostaccio, Rasha Ahmed Hanafy Bayomi, Sunghoon Lee, Gaetano Marrocco, Takao Someya, Cecilia Occhiuzzi

**Affiliations:** 1 Radio6ense S.r.l., Via Della Tenuta di Torrenova 142, 00133 Rome, Italy; barba@radio6ense.com; 2 DICII-Tor Vergata University of Roma, Via del Politecnico 1, 00133 Rome, Italy; mostaccio@ing.uniroma2.it (A.M.); gaetano.marrocco@uniroma2.it (G.M.); 3 RIKEN-Institute of Physical and Chemical Research, Saitama 351-01, Japan; rasha.bayomi@riken.jp (R.A.H.B.); sunghoon.lee@riken.jp (S.L.);

**Keywords:** epidermal electronics, tattoo antenna, UHF RFID, substrate-free conductor, gold nanomesh

## Abstract

Substrate-free epidermal antennas promise imperceptible and long-term wearable sensing, yet their electromagnetic performance is fundamentally constrained by the properties of ultrathin conductors. In this work, gold nanomesh is employed for the first time as the radiating conductor of a substrate-free epidermal tattoo antenna operating in the UHF RFID band. Owing to its RF-thin nature, the nanomesh behavior is governed by sheet resistance rather than skin-depth effects, imposing a strict upper bound on achievable radiation efficiency. By combining surface-impedance modeling, full-wave simulations, and on-body experiments, we demonstrate that ohmic losses set a geometry-independent limit on the realized gain of on-skin antennas. An inductively coupled loop architecture is optimized to approach this bound while ensuring mechanical robustness and impedance stability. Measurements on phantoms and human subjects confirm the predicted performance limits within a few decibels, enabling reliable UHF RFID read ranges up to 30–40 cm under standard regulatory constraints.

## 1. Introduction

The skin represents a chemically, mechanically, and physiologically dynamic interface that encodes information related to temperature, hydration, biomechanical activity, and biochemical composition. Accessing these signals in a noninvasive and continuous manner has driven the development of *skin-integrated electronics,* i.e., ultrathin and compliant systems capable of operating directly at the epidermis while accommodating natural deformation, perspiration, and long-term wear [1,2,3,4,5,6]. Within this framework, electronic tattoos (e-tattoos) [7,8,9] rely on filamentary or membrane-like conductive layouts that laminate conformally onto the skin [10], offering minimal perceptibility, high conformability [11], and gas permeability. These characteristics have enabled long-term epidermal sensing of biopotentials [12], sweat composition, hydration, and motion-related quantities [13].

Among the materials explored for epidermal conductors, metallic nanomeshes have recently emerged as a particularly promising solution. Gold nanomesh (G-nm) consists of a porous metallic network obtained by depositing gold onto an electrospun polyvinyl alcohol (PVA) scaffold, which is subsequently removed after transfer onto the skin, leaving a freestanding conductive mesh [14,15]. Owing to its open morphology, G-nm combines electrical conductivity with gas permeability, stretchability, and long-term biocompatibility [15]. These properties have supported its application in on-skin strain sensing, electrophysiology, and biopotential monitoring [14,16], positioning gold nanomesh as a viable substrate-free platform for epidermal electronics.

While skin-mounted sensors can locally acquire physiological information, their practical deployment critically depends on the availability of reliable data-transfer mechanisms. For epidermal systems intended for long-term and unobtrusive use, wireless and batteryless communication is highly desirable to avoid bulky power sources, reduce maintenance, and preserve mechanical compliance. In this context, Ultra-High Frequency (UHF) radio-frequency identification (RFID), operating in the 860–960 MHz band, represents an attractive enabling technology [17]. Passive UHF RFID systems support battery-free operation through electromagnetic energy harvesting, are compatible with sensing-oriented microchips [18], and have demonstrated robustness in short- to medium-range body-centric links. As a result, UHF RFID has been successfully applied to epidermal temperature monitoring [19], electrochemical sweat sensing [20], and skin-mounted wireless patches [18], confirming its feasibility on human subjects.

A key challenge in epidermal UHF RFID systems lies in the design of on-skin antennas capable of maintaining stable impedance matching and sufficient radiation efficiency in the presence of highly lossy biological tissues [17]. Most reported implementations rely on conductors and fabrication processes derived from flexible or wearable electronics, including copper or aluminum traces on polyimide substrates [21], conductive filaments supported by polyurethane layers [22], and metallic foils transferred onto silicone elastomers. Laser-induced graphene (LIG), obtained by photothermal conversion of polyimide, has also been explored as an emerging conductor for conformal epidermal UHF RFID antennas [23]. Despite their effectiveness, these substrate-supported solutions impose intrinsic limitations when targeting true epidermal integration. Polymeric or elastomeric substrates hinder breathability, promote moisture accumulation, and introduce mechanical mismatch with the viscoelastic skin, which can lead to stress-induced delamination and degradation of antenna–body coupling during prolonged wear [22].

These considerations motivate the exploration of *substrate-free* conductors that can adhere directly to the skin while sustaining efficient electromagnetic radiation. Within this perspective, gold nanomesh provides a compelling opportunity to merge skin-integrated electronics with batteryless UHF RFID communication. Preliminary numerical investigations have suggested the feasibility of substrate-free G-nm tattoo antennas operating in the UHF band [24]. In contrast, for high-frequency (HF) RFID at 13.56 MHz, the same material was shown to be severely constrained, requiring an impractically large thickness (>20 μm) to meet resonance and quality-factor requirements [24]. These results identify the UHF band as the most suitable operational range for gold-nanomesh-based epidermal antennas.

Despite these promising indications, a systematic and quantitative assessment of how the sheet resistance and sub-skin-depth behavior of gold nanomesh affect the radiation efficiency, impedance stability, and achievable read range of on-skin UHF antennas is still missing. In particular, the upper bounds of electromagnetic performance attainable with substrate-free, RF-thin conductors on different body regions remain largely unexplored. The objective of this work is therefore to determine and experimentally approach the upper limits of the electromagnetic performance of gold-nanomesh loop antennas for UHF RFID applications. This is achieved through a combined approach involving theoretical modeling, full-wave simulations, and experimental validation on phantom and human subjects.

The remainder of the paper is organized as follows. Section 2 discusses the electromagnetic properties of gold nanomesh conductors and their operation in the sub-skin-depth regime. Section 3 recalls the fundamentals of UHF RFID relevant to epidermal applications and introduces the performance metrics adopted throughout the paper, together with the main challenges of on-skin tags. Section 4 presents the design of the epidermal loop antenna and analyzes its electromagnetic performance as a function of geometric and material parameters. Finally, Section 5 reports the experimental characterization of the gold nanomesh material and the validation of prototype tattoo antennas in phantom and on-body configurations.

## 2. The Gold Nanomesh Conductor

**Figure 1:**
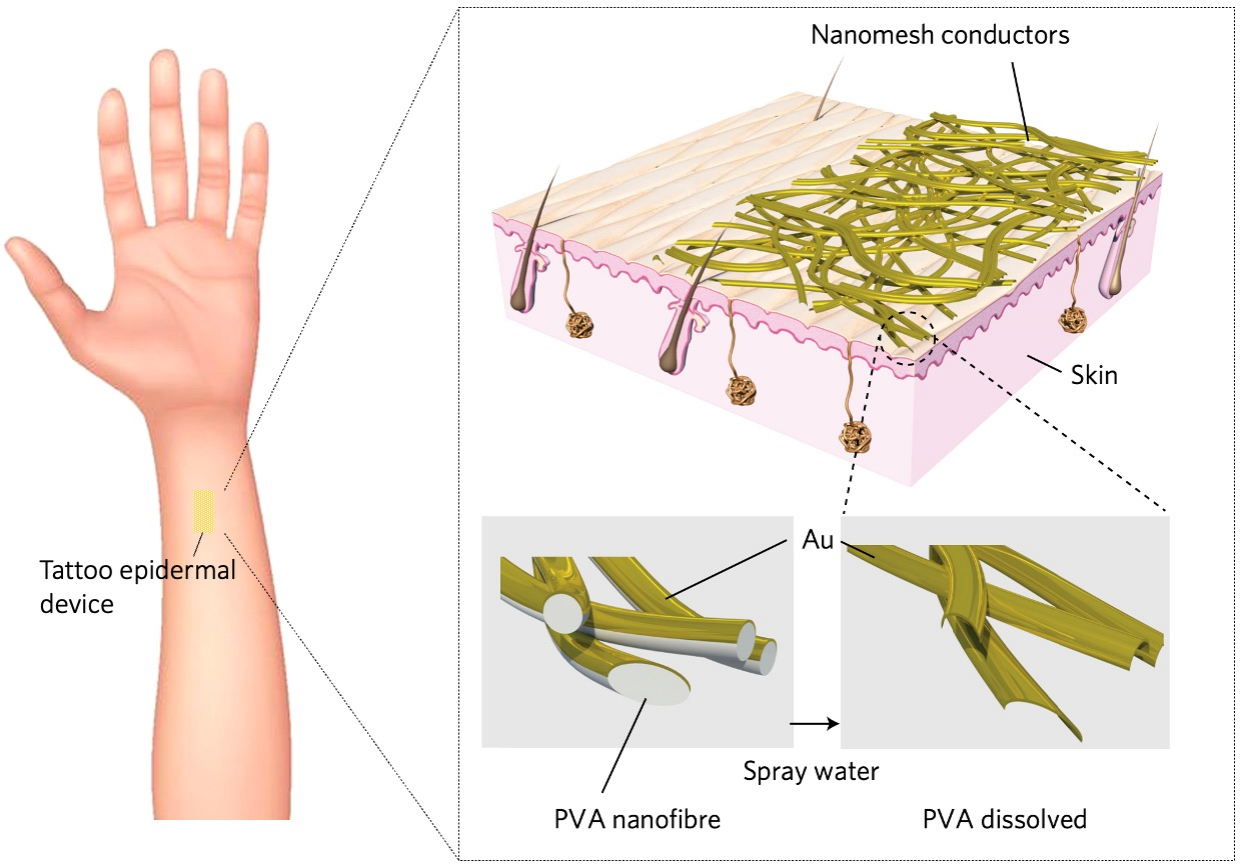
Schematic illustration of the gold nanomesh conductor applied directly on human skin. After deposition, the PVA nanofiber scaffold is removed by a light water spray, resulting in a conformal gold nanomesh that remains firmly attached to the skin surface. Adapted from [14].

**Figure 2:**
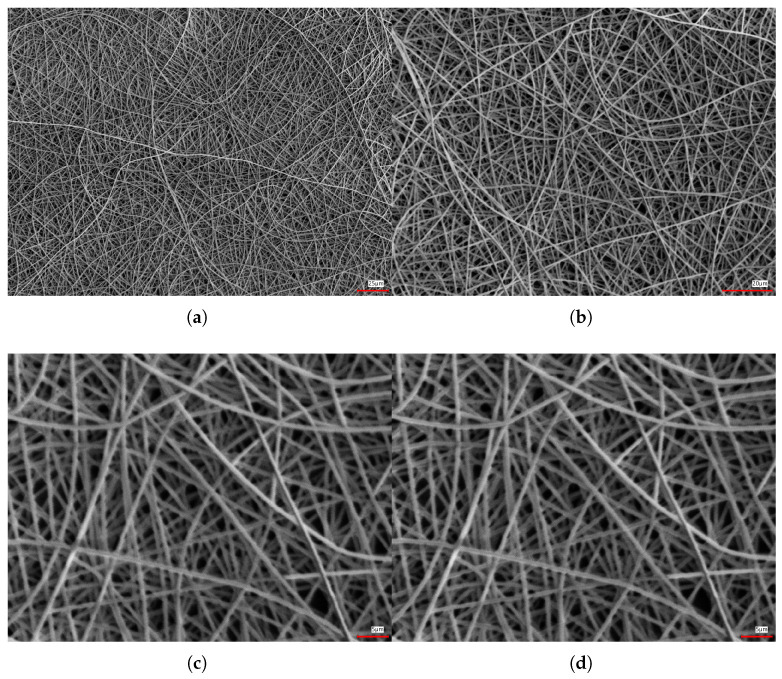
Scanning electron microscopy (SEM) images of the gold nanomesh (G-nm) at different magnifications, highlighting its interconnected, porous network morphology across length scales: (**a**) low-magnification overview (scale bar: 25 μm); (**b**) intermediate magnification (scale bar: 20 μm); (**c**,**d**) high-magnification views showing the local mesh structure and interconnections (scale bars: 5 μm).

The G-nm conductor is fabricated by electrospinning a polyvinyl alcohol (PVA) nanofiber network onto a temporary substrate, followed by sputter deposition of a thin gold film, typically 100–120 nm thick. This process yields a porous metallic structure that is subsequently transferred onto the target surface, such as human skin. Upon exposure to water, the sacrificial PVA scaffold dissolves, releasing a freestanding gold mesh that conforms intimately to the epidermal microrelief without the need for adhesives (Figure 1). Owing to its intrinsic porosity, the resulting conductor is lightweight, breathable, and mechanically compliant. The interconnected filamentary network (Figure 2) enables gas and moisture permeability, mitigating sweat accumulation and reducing mechanical mismatch with the viscoelastic skin, thereby ensuring stable epidermal contact under bending, stretching, and shear deformations during prolonged wear.

From an electrical standpoint, gold nanomesh is characterized by a DC sheet resistance $R_{s}$, which depends on the effective conductor thickness *t* and conductivity σ:

(1) $$R_{s}=\frac{1}{\sigma t}.$$

Due to surface scattering, grain-boundary effects, and partial filling of the conductive area, the effective conductivity of the nanomesh is lower than that of bulk gold. As a result, typical sheet resistance values are on the order of a few Ω/sq, even for nominal thicknesses comparable to continuous metallic films [15].

At radio frequencies, current distribution in conductive materials is governed by the skin effect, which confines currents within an effective penetration depth

(2) $$\delta \left(f\right)=\sqrt{\frac{1}{\pi \mu f\sigma }}$$

where μ is the magnetic permeability, *f* the operating frequency, and σ the electrical conductivity. Unlike continuous metallic films, gold nanomesh operates in the *RF-thin* regime, in which the physical thickness of the conductor is much smaller than the electromagnetic skin depth [25]. Under these conditions, the current is distributed across the entire cross section of the conductor rather than being confined near the surface, and the surface impedance becomes frequency-independent, coinciding with the DC sheet resistance $R_{s}$ [24]. Because of the extremely small effective thickness of the nanomesh, this resistance can be relatively high, making ohmic losses the dominant factor limiting radio-frequency performance.

This behavior is particularly critical in passive UHF RFID systems, where antenna efficiency and achievable read range are strongly constrained by series losses. Consequently, the electromagnetic performance of gold-nanomesh-based epidermal antennas is fundamentally bounded by the material sheet resistance, motivating the upper-bound analysis developed in the following sections.

## 3. RFID Technology for Epidermal UHF Applications

Passive UHF RFID is an enabling technology for batteryless epidermal sensing, allowing ultra-lightweight devices to be remotely powered and interrogated through electromagnetic backscattering. An RFID system consists of two main components: (i) the *reader*, which emits an unmodulated continuous wave (CW) and processes the received backscattered signal, and (ii) the *tag*, which integrates a radiating element, an impedance-matching network, and an integrated circuit (IC) for energy harvesting and data modulation [26].

In passive UHF RFID systems, overall performance is primarily constrained by the *forward link* (reader-to-tag), since tag activation requires the harvested power to exceed the IC sensitivity threshold. This aspect is particularly critical for epidermal applications, where the electromagnetic interaction with the human body introduces strong attenuation and limits the power available at the tag. Once activated, the tag-to-reader backscatter link generally provides sufficient margin at the short read distances of interest and is therefore not the dominant limiting factor considered in this work.

### 3.1. RFID Tag Architecture for Epidermal Devices

The radiating element of a passive epidermal RFID tag, here implemented as a gold-nanomesh loop, converts the incident electromagnetic field into RF voltage and current. Its performance is strongly affected by body loading and material resistivity, as the high permittivity and conductivity of skin, fat, and muscle layers cause substantial absorption and reduce radiation efficiency [17]. Moreover, for RF-thin conductors such as gold nanomesh, ohmic losses associated with the sheet resistance further constrain the achievable gain.

Impedance matching between the antenna and the IC is achieved through an inductively coupled adapter, which conjugates the antenna input impedance to the IC admittance. This non-galvanic configuration mitigates contact-related losses and provides stable matching despite variations in tissue composition, hydration, and antenna placement on the body. Such stability is essential in epidermal scenarios, where even small impedance detuning can significantly reduce the harvested power.

The RFID microchip is a passive UHF transponder characterized by an input admittance $Y_{\mathrm{IC}}=G_{\mathrm{IC}}+jB_{\mathrm{IC}}$, a power sensitivity $P_{\mathrm{th}}$, and a load-modulation mechanism used to encode information onto the backscattered field. During operation, the chip alternates between two impedance states to modulate the radar cross section of the tag once sufficient power has been harvested.

### 3.2. Forward Link and Activation Condition

Following [27], the power $P_{R\to T}$ delivered to the tag antenna can be expressed as

(3) $$P_{R\to T}={\left(\frac{\lambda_{0}}{4\pi r}\right)}^{2}G_{R}\left(\theta ,\phi \right)\;G_{T}\left(\theta ,\phi \right)\;\chi \;P_{\mathrm{in}}\;\tau ,$$

where θ and ϕ are the angles on the E-plane and H-plane respectively, *r* is the reader-tag distance, $G_{R}$ and $G_{T}$ are the gains of the reader and tag antennas, χ accounts for polarization mismatch, and τ is the power transmission coefficient defined as

(4) $$\tau =\frac{4R_{\mathrm{IC}}R_{A}}{|Z_{A}+Z_{\mathrm{IC}}{|}^{2}}.$$

with $Z_{\mathrm{IC}}=Y_{\mathrm{IC}}^{-1}$ IC input impedance and $Z_{A}=R_{A}+jX_{A}$ input impedance of the antenna.

Tag activation occurs when the harvested power exceeds the IC sensitivity threshold:

(5) $$P_{R\to T}\geq P_{\mathrm{th}}.$$

From this condition, the maximum achievable read distance can be derived as

(6) $$r_{\max}=\frac{\lambda_{0}}{4\pi }\sqrt{\frac{G_{R}G_{T}\chi \;P_{\mathrm{in}}\;\tau }{P_{\mathrm{th}}}},$$

which represents the UHF-RFID counterpart of Friis’ transmission formula. This expression highlights the central role of the *realized gain* of the tag antenna,

(7) $${\hat{G}}_{T}=G_{T}\;\tau ,$$

as the key parameter governing tag activation and read range.

For epidermal antennas [17], the realized gain is typically low (−10 to −20 dBi). This severe reduction arises from the combined effect of electromagnetic absorption in the skin–fat–muscle layers, ohmic dissipation in ultrathin RF-thin conductors, and impedance mismatch induced by tissue-related detuning. As a result, the forward link constitutes the dominant performance bottleneck in epidermal UHF RFID systems and ultimately bounds the achievable read range.

## 4. Antenna Design

The radiating element adopted in this work is a circular loop made of gold nanomesh placed directly on the epidermis. The loop geometry is selected because, for a given enclosed area, it maximizes radiation efficiency and minimizes the impact of conductor losses, as demonstrated in previous studies on epidermal UHF antennas under strong body loading [17].

A direct galvanic connection between the nanomesh and the RFID microchip is avoided for both mechanical and electromagnetic reasons. Substrate-free conductors are prone to local damage or delamination under shear stresses, making soldering unreliable.

Moreover, as observed for laser-induced graphene epidermal antennas [28], the differential modes generated by circular paths of the currents introduces severe degradations of the radiation efficiency in the case of lossy conductors. To overcome these limitations, the antenna is excited through inductive coupling [29] using a smaller secondary loop (adapter) hosting the RFID IC and implemented on a 0.04 mm thick Polyethylene Terephthalate (PET) substrate ($\varepsilon_{r}=3.2,\;\tan\delta =0.014\;\mathrm{at}\;1\;\mathrm{MHz}$). This loop–match architecture ensures (i) reproducible impedance matching; (ii) elimination of contact-related loss mechanisms; (iii) independent fabrication or replacement of the gold-nanomesh radiating element; and (iv) stable response of the G-nm radiator w.r.t. the position of the exciter, thereby providing a certain degree of tolerance in the manufacturing process. The resulting architecture consists of the gold nanomesh loop acting as the sole radiating structure and an inductively coupled adapter providing a transformer-like interface that conjugates the antenna input impedance to that of the RFID chip. In this study, the antenna is matched to the Magnus S3 transponder (Axzon Inc., Austin, TX 78730 (USA)), a passive UHF IC with a power sensitivity of $P_{C}=-13.6\;\mathrm{dBm}$ and a nominal input admittance of $Y_{IC}=0.482+j\omega C_{IC}\;\mathrm{mS}$ at 900 MHz. The chip includes an auto-tuning mechanism that dynamically adjusts its internal capacitance to preserve impedance matching under varying antenna conditions, and it integrates a temperature sensor with a resolution of ±0.25 °C, making it suitable for epidermal sensing applications.

**Figure 3:**
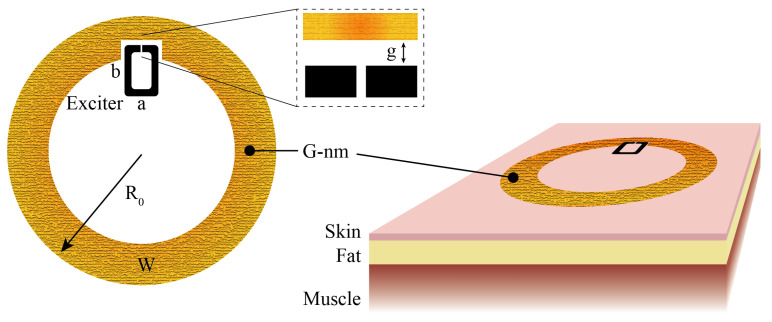
Layout of the epidermal antenna including the gold nanomesh main radiator and the aluminum exciter loop (black) hosting the RFID IC. Positioning on the three-layer human phantom on the right. Numerical simulations performed through CST Microwave Studio.

**Figure 4:**
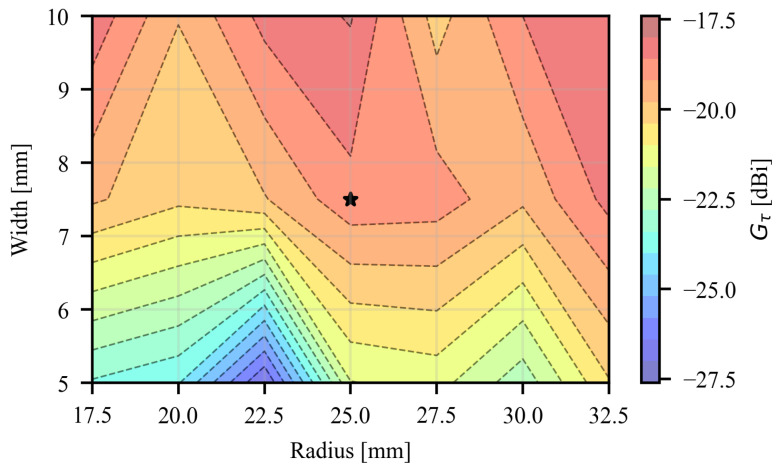
Contour plot of the antenna realized gain as a function of the loop outer radius and trace width. The marker indicates the selected configuration.

The gold nanomesh was modeled as a thin resistive sheet with a surface resistance of $R_{s}$ = 1 Ω/sq, consistent with literature values for a single 200 nm layer [15]. For taking into account the on-skin application, the antenna was simulated as being applied at the center of a multilayer human phantom (20 × 20 × 5.1 cm^3^) consisting of a 1 mm-thick skin layer ($\varepsilon_{r}=43.8,\;\tan\delta =0.4\;\mathrm{at}\;868\;\mathrm{MHz}$), a 1 cm-thick fat layer ($\varepsilon_{r}=5.5,\;\tan\delta =0.2\;\mathrm{at}\;868\;\mathrm{MHz}$), and a 4 cm-thick muscle layer ($\varepsilon_{r}=54.4,\;\tan\delta =0.3\;\mathrm{at}\;868\;\mathrm{MHz}$), as in Figure 3. A parametric analysis was carried out by varying both the outer diameter of the radiating loop, $R_{0}$, and the width of the conductive trace, *W*, in order to explore their combined effect on the antenna’s realized gain. For each combination of $R_{0}$ and *W*, the geometrical parameters of the smaller exciter loop, *a* and *b*, were varied to maximize the power transfer coefficient in Equation (4). Results are reported in Figure 4. As expected for epidermal antennas made by lossy conductors, a non-monotonic trend was observed: the radiation performance does not increase indefinitely with size (i.e., the outer radius) but exhibits a clear optimum, beyond which additional enlargement is counterproductive due to increased ohmic and reactive losses. The trace width is the most impactful parameter, providing a 3 dB improvement in the realized gain by enlarging the trace width from 5 mm to 10 mm. This behavior is in agreement with previous findings on epidermal antennas made from continuous conductors, where similar trade-offs between radiation efficiency and conductor loss have been reported [17]. It is worth mentioning that excessively wide traces may compromise the mechanical robustnessnof the conductor, whereas thin traces reduce the radiation performance of the antenna. Accordingly, a mechanical-electromagnetic trade-off was found for $R_{0}$ = 25 mm and *W* = 7.5 mm, which corresponds to a realized gain $G_{\tau }$ = −18.8 dBi (i.e., approximately 2 dB lower than the maximum value equal to −16.5 dBi).

**Figure 5:**
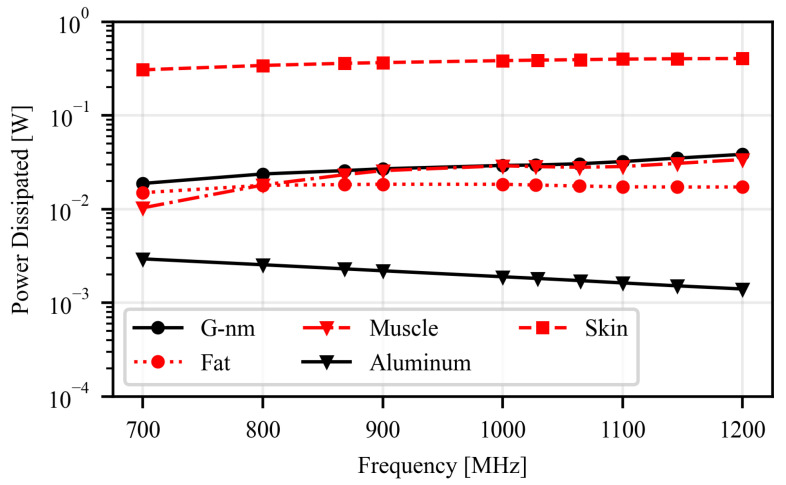
Power dissipated by ohmic and dielectric losses in the human tissues and antenna elements, referred to an input power of 0.5 W.

Such a value is consistent with the strong loss mechanisms introduced by the on-body operating scenario. In particular, the radiation efficiency is mainly limited by power dissipation in the human tissues (Figure 5), which accounts for approximately 83% of the input power, with the skin being the dominant contributor due to its close proximity to the radiating element. Additional losses are introduced by the finite conductivity of the G-nm conductor, which further increase the overall dissipated power by about 5%. As a result, only a small fraction of the input power is effectively radiated. Under standard operating conditions (3.2 W EIRP and conjugate matching), the antenna is nevertheless expected to support read distances of up to 60 cm, thereby confirming its suitability for short-range epidermal sensing systems.

**Figure 6:**
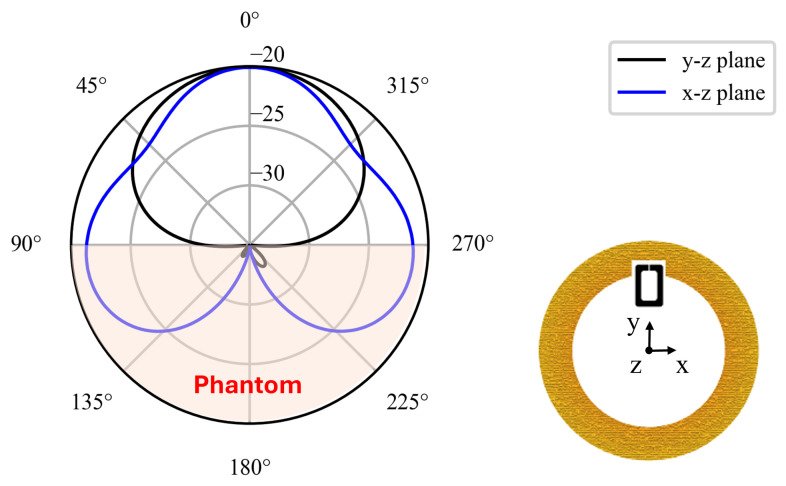
Radiation pattern of the epidermal antenna on the x-z and y-z planes according to the coordinate system in the inset.

Figure 6 reports the radiation pattern of the antenna on two orthogonal planes referred to the coordinate system shown in the inset. The presence of the human body strongly affects the pattern of the loop antenna by attenuating the radiation toward the phantom and producing a broad maximum toward free space (front hemisphere, $\theta \approx 0^{\circ }$).

**Figure 7:**
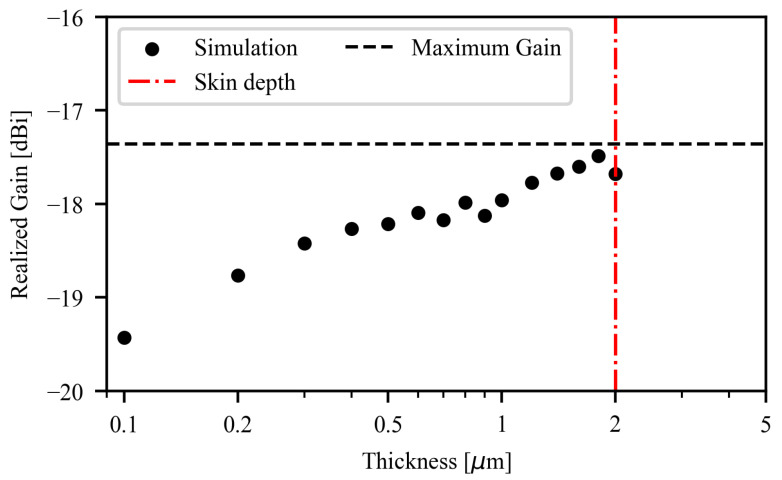
Realized gain of the epidermal loop as a function of the gold nanomesh thickness. The red dashed vertical line indicates the penetration depth, whereas the black horizontal line reports the maximum achievable gain with bulk-gold conductor.

Upon assessing the optimal size of the radiating loop, the impact of the conductor’s sheet resistance was investigated. The thickness of the gold nanomesh conductor was varied between 100 nm and 2 μm, corresponding to $0.2<R_{s}<10\;\Omega /\mathrm{sq}$. It is worth mentioning that the lowest sheet resistance corresponds to the skin depth limit. Hence the condition $R_{s}=0.2\;\Omega /\mathrm{sq}$ is electromagnetically equivalent to bulk metallic conductor. Simulation results (Figure 7) show that the realized gain of the gold nanomesh loop, by assuming perfect impedance matching, ranges from −19.4 dBi to −17.3 dBi, thus indicating an improvement of only 2.1 dB when considering a metallic loop of the same geometry. Such a difference is considered acceptable in most passive UHF RFID applications.

## 5. Experimental Characterization

Building upon the numerical analysis presented in the preceding sections, a comprehensive experimental campaign was undertaken to assess the feasibility of employing gold nanomesh conductors in substrate-free UHF RFID tattoo antennas. While simulations provided insight into the electromagnetic behavior of nanomesh-based radiators, highlighting the impact of sheet resistance, conductor thickness, and antenna geometry, experimental validation remains essential to confirm the accuracy of the simulation model, verify the consistency of the fabrication process, and quantify the antenna performance under realistic operating conditions.

### 5.1. Material Characterization

**Figure 8:**
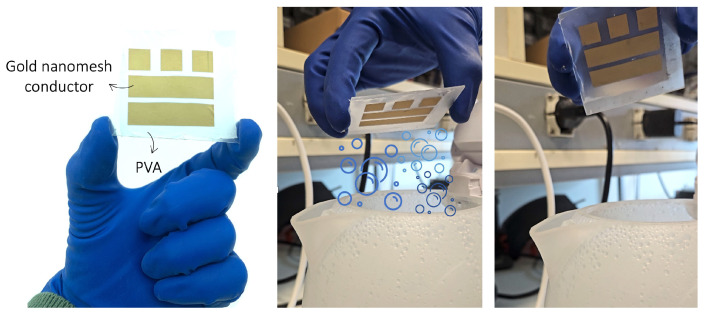
Gold nanomesh samples supported on a PVA scaffold (**left**), transferred onto a glass slide where the PVA matrix is removed through water vapor exposure (**center**), and the resulting bare gold nanomesh remaining on the slide after complete dissolution (**right**).

To experimentally assess the electrical properties of the gold nanomesh conductor, six rectangular samples (S1–S6), each with dimensions of 1 cm × 4 cm and 100–120 nm thickness, were prepared on a PVA nanofiber scaffold and subsequently transferred onto glass substrates. After transfer, the PVA support was removed by exposure to water vapor, leaving the free-standing metallic mesh firmly adhered to the glass surface and suitable for measurement (Figure 8).

The sheet resistance of each sample was then quantified using a Four-Point Probe system (Ossila), which applies a controlled current through the outer probes while sensing the voltage drop across the inner probes, thereby eliminating contact resistance contributions and yielding an accurate estimate of the intrinsic material resistance. The measured sheet resistance values for the six samples are reported in Table 1. Across the entire set, the gold nanomesh exhibited an average sheet resistance of $R_{s}=5.2\pm 1.9$ Ω/sq, demonstrating good repeatability of the fabrication process.

### 5.2. Epidermal UHF Antenna Characterization

After characterizing the electrical properties of the G-nm material, epidermal loop antennas were fabricated to evaluate the suitability of this ultrathin conductor for UHF RFID on-body operation. The study aimed to (i) compare the G-nm antenna with an identical bulk gold version and (ii) assess the performance of the tattoo-like antenna when deposited on different anatomical sites. The antenna design was derived from the measured sheet resistance of approximately 5 Ω/sq. Following the simulation guidelines of Section 4, a circular loop with a 50 mm outer diameter and a 7.5 mm trace width was selected.

**Figure 9:**
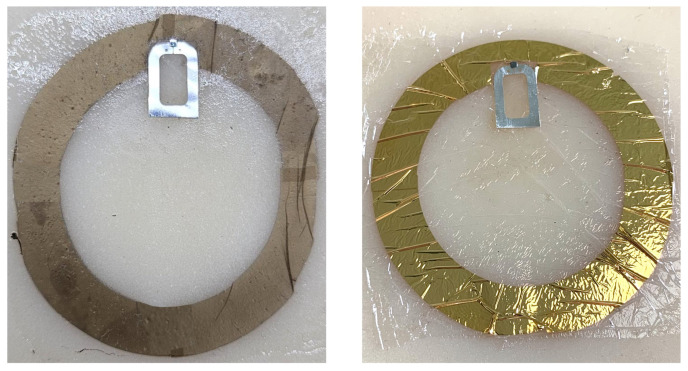
Prototypes of the G-nm loop antenna (**left**) and its bulk-gold counterpart **(right**), both applied to the phantom.

Two versions of the radiating loop antenna were prototyped, namely, a gold nanomesh loop fabricated on a PVA scaffold and a reference loop fabricated using a continuous gold layer deposited on a parylene film (Figure 9), to serve as a material performance benchmark. Both loop prototypes were inductively coupled to a metallic exciter equipped with the Magnus S3 RFID chip mounted on a flexible polyethylene terephthalate (PET) substrate, acting as a reusable module without soldered connections.

**Figure 10:**
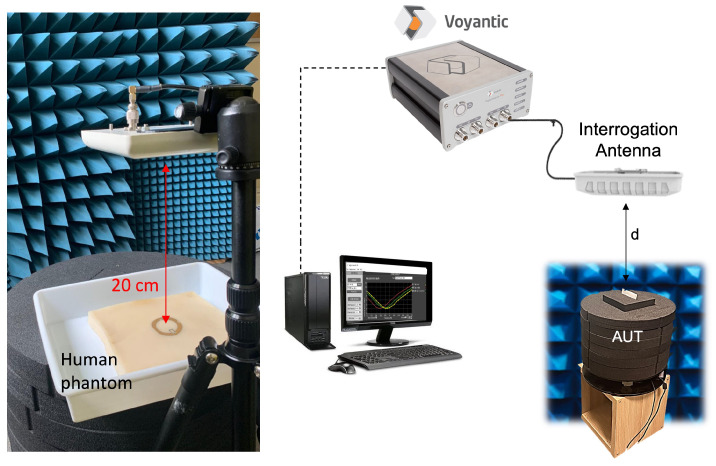
Measurement setup inside the semi-anechoic chamber: the G-nm antenna prototype applied to the human phantom (**left**), and the complete acquisition system **(right**). The system includes the antenna under test (AUT) positioned at a distance d from the interrogation antenna, which is connected to the Voyantic Tagformance Pro measurement unit and operated through its dedicated PC software (Vr. 13.8).

All measurements were carried out in a semi-anechoic chamber using a Voyantic Tagformance system setup with a 7 dBic circularly polarized antenna (Figure 10), and employing the turn-on power method [27] to extract the realized gain and theoretical read range.

#### 5.2.1. Phantom-Based Testing

**Figure 11:**
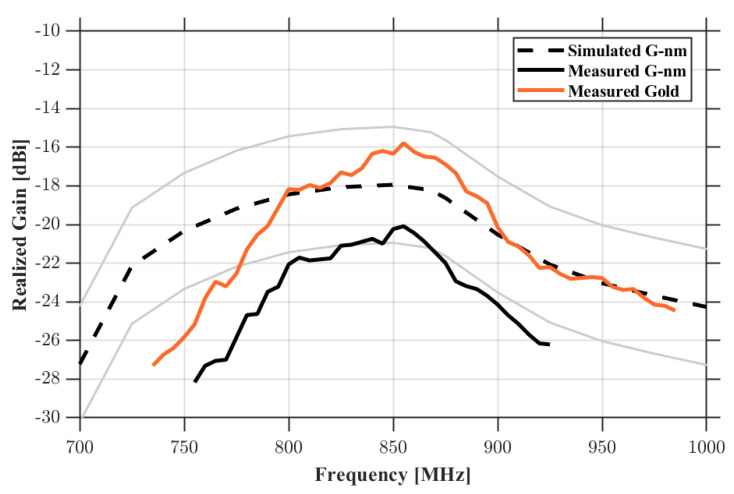
Realized gain of the loop antenna on a human phantom: simulated response of the gold nanomesh loop antenna (including a ±3 dB margin), measured performance of the gold nanomesh prototype, and measured performance of the corresponding bulk gold loop.

The first experimental session was carried out using a stratified human-tissue phantom (30 × 30 × 8 cm^3^) designed to replicate the dielectric properties of skin, fat, and muscle in the UHF band. The gold nanomesh loop antenna was placed at the center of the phantom surface, at a distance of 20 cm from the interrogation antenna (Figure 10), and the PVA scaffold was dissolved in situ using water vapor to achieve direct contact between the nanomesh and the phantom surface. Figure 11 reports the measured realized gain of the G-nm antenna alongside the full-wave simulation results, assuming an uncertainty margin of 3 dB as acceptable. The two curves show very good agreement, with deviations consistently below 2.5 dB within the European UHF RFID band (865–868 MHz) of interest. When the G-nm loop is replaced by the bulk-gold prototype and measured under identical conditions, the realized gain increases by approximately 3 dB (Figure 11). This difference is consistent with the expected impact of the higher sheet resistance of the gold nanomesh and corresponds to a reduction in read range by roughly a factor of $\sqrt{2}$.

Overall, these results confirm the validity of the electromagnetic model adopted for the gold nanomesh conductor and demonstrate that it can effectively operate as a radiating element for substrate-free epidermal antennas.

#### 5.2.2. On-Body Testing

**Figure 12:**
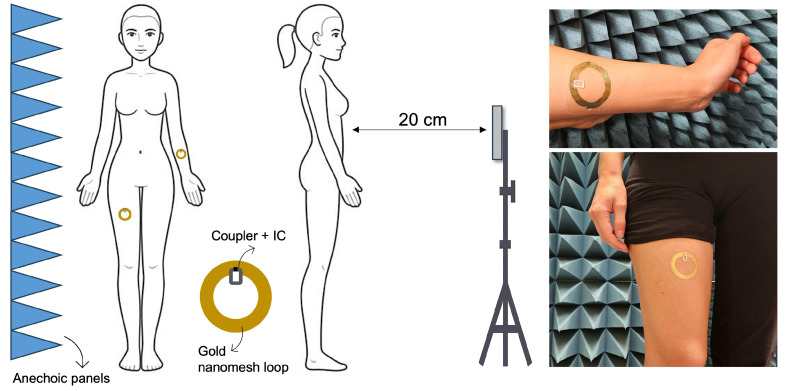
Measurement setup of the G-nm antennas applied to the body inside the semi-anechoic chamber, with the interrogation antenna positioned 20 cm away (**left**). G-nm loop antennas placed on the forearm and thigh of a human volunteer (**right**).

The communication performance of the gold nanomesh loop prototypes were subsequently evaluated by placing them directly on the skin of a human subject (BMI = 18.5), using the standard PVA-dissolution transfer process. Two anatomical sites were selected for testing, namely the inner forearm (representative of a relatively flat, low-loss region) and the thigh (characterized by greater curvature and higher muscle content), as displayed in Figure 12.

**Figure 13:**
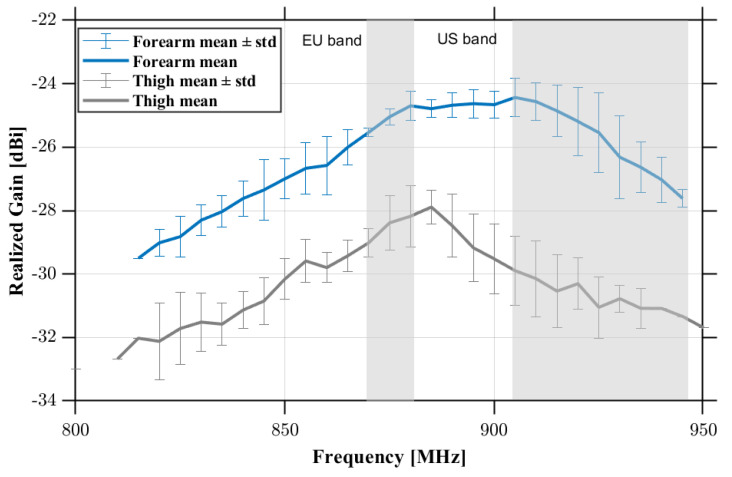
Average realized gain ± standard deviation over the five independent measurements performed on the volunteer’s arm and thigh using the G-nm loop antennas.

During all measurements, the interrogation antenna was positioned 20 cm from the epidermal loop, oriented perpendicularly to the skin surface, while its height was adjusted to always stay in line with the epidermal tags under test. At the adopted interrogation distance, the proposed on-body measurements are safe from a human exposure standpoint [30,31]. For each anatomical site, five independent acquisitions were carried out. The results, shown in Figure 13, indicate that the realized gain on the forearm is approximately 3 dB higher than that measured on the thigh, in agreement with literature evidences on epidermal RFID antennas showing site-dependent performance variations across different body locations [22,32]. Over the European UHF RFID band (865–868 MHz), the average realized gain is −26±0.6 dBi on the forearm and −29.4±0.5 dBi on the thigh. Assuming the European regulatory limit of 3.2 W EIRP, these values correspond to maximum theoretical read ranges of ~37 cm and ~25 cm respectively. The antenna exhibits comparable behavior in the North American RFID band (902–928 MHz). The average realized gain in this band is −25.2±0.9 dBi for the forearm and −30.5±0.9 dBi for the thigh (within approximately 1 dB of the values measured in the EU band) confirming that this gold nanomesh antenna provides stable electromagnetic performance across both major UHF allocations.

## 6. Conclusions

This work has established, for the first time, a quantitative upper bound on the electromagnetic performance of substrate-free epidermal tattoo antennas based on gold nanomesh conductors operating in the UHF RFID band. By experimentally employing gold nanomesh as the radiating element of a tattoo antenna, this study demonstrates both the feasibility and the intrinsic limitations of RF-thin, substrate-free metallic conductors for epidermal wireless systems. Through a combined theoretical, numerical, and experimental analysis, it has been shown that the RF behavior of G-nm is governed by its sheet resistance rather than by classical skin-depth effects. As a consequence, ohmic losses impose a strict and largely geometry-dependent bound on the achievable realized gain of on-skin antennas. While appropriate antenna geometries and impedance-coupling strategies—such as inductively coupled loop architectures—can be optimized to approach this limit, they cannot overcome it.

Experimental results obtained on phantoms and human subjects confirm the predicted performance bounds within a few decibels, yielding stable and reproducible UHF RFID read ranges up to 30–40 cm under standard regulatory constraints. These findings indicate that gold nanomesh enables reliable substrate-free epidermal RFID operation, while simultaneously clarifying the performance ceiling associated with its RF-thin nature. In real-world deployments, the epidermal tag can be interrogated using standard UHF RFID readout architectures depending on the monitoring scenario: fixed reader and antennas integrated in the environment [31] or wearable readers [33] enabling close-range continuous logging. Conversely, sporadic readout can be performed on demand using handheld readers [34] or RFID gates/portals for rapid screening workflows [35].

Several open challenges remain and define clear directions for future research. First, a systematic investigation of inter-subject and intra-subject variability is required to quantify the impact of anatomical differences, skin composition, hydration level, and placement location on antenna performance. Second, long-term studies are needed to assess the mechanical durability and environmental stability of gold nanomesh under prolonged wear, including the evolution of its electrical and RF properties under repeated deformation, perspiration, and exposure to external agents. Finally, the integration of sensing functionalities represents a promising direction, enabling multifunctional epidermal tags that combine wireless identification with physiological or biochemical sensing while operating within the established electromagnetic performance bounds.

## Figures and Tables

**Table 1:** Sheet resistance [Ω/sq] values for each G-nm sample $S_{i}$.

	S1	S2	S3	S4	S5	S6
$R_{s}$ [Ω/sq]	7.92	3.55	4.73	4.23	4.25	6.74

## Data Availability

The data presented in this study are available on request from the corresponding author.
